# Side effects of the leukotriene receptor antagonists in asthmatic children

**DOI:** 10.1186/1939-4551-8-S1-A6

**Published:** 2015-04-08

**Authors:** Demet Can, Semiha Bahceci Erdem, Hikmet Tekin Nacaroglu, Canan Sule Unsal Karkiner, Ilker Gunay

**Affiliations:** 1 Behcet Uz Children Hospital, Turkey

## Background

Leukotriene receptor antagonists (LTRAs) are drugs which have been widely used more than ten years. As the use of LTRAs increases, our knowledge with respect to their side effects increases as well.

Objective: Our study was aimed to evaluate the observed side effects of LTRAs used in patients with astma.

## Methods

1024 patients who were only treated with LTRAs owing to astma or early wheezing were included in the study for a five-year period. The observed side effects of LTRAs in these patients were retrospectively investigated. The side effects were divided into two parts as psychiatric and non- psychiatric.

## Results

It was found out that 67.5% of 41 (4%) patients in whom side effects were observed was male and their average age was 6.5. The rate of patients with asthma was 63.41% and it was 36.58% for patients with early wheezing. It was determined that sex, age and diagnosis (early wheezing or asthma) of the patients were ineffective in the emergence of side effects. The average period for the emergence of side effects was the fisrt month. It was observed that hyperactivity was the most frequently seen psychiatric side effect and abdominal pain was the non-psychiatric side effect.

## Conclusions

The side effects of LTRAs were common in children. Therefore, patients must be informed at the beginning of the treatment and they must be evaluated at certain intervals.

